# Fabrication and Properties of High-Efficiency Perovskite/PCBM Organic Solar Cells

**DOI:** 10.1186/s11671-015-1020-2

**Published:** 2015-08-05

**Authors:** Lung-Chien Chen, Jhih-Chyi Chen, Cheng-Chiang Chen, Chun-Guey Wu

**Affiliations:** Department of Electro-Optical Engineering, National Taipei University of Technology, 1, Section 3, Chung-Hsiao E. Road, Taipei 106, Taiwan; Research Center for New Generation Photovoltaics, National Central University, Taoyuan, 32001 Taiwan

**Keywords:** XRD, Absorption, Organic solar cells, CH_3_NH_3_PbI_3_ perovskite

## Abstract

This work presents a CH_3_NH_3_PbI_3_/PCBM organic solar cell. Organic PCBM film and CH_3_NH_3_PbI_3_ perovskite film are deposited on the PEDOT:PSS/ITO glass substrate by the spin coating method. The performance of the organic solar cells was observed by changing the thickness of CH_3_NH_3_PbI_3_ perovskite. The thickness of a perovskite film can affect the carrier diffusion length in a device that strongly absorbs light in the red spectral region. The short-circuit current density and the power conversion efficiency were 21.9 mA/cm^2^ and 11.99 %, respectively, for the sample with 210-nm-thick CH_3_NH_3_PbI_3_ perovskite active layer.

## Background

Perovskite solar cell has attracted considerable attention because of its unique properties and potential applications. As the hybrid organic/inorganic lead halide perovskite (e.g., CH_3_NH_3_PbX_3_, X = I, Cl, Br) materials, perovskite has a high absorption coefficient, long hole–electron diffusion length (~0.1–1 μm), tunable band gaps, and good carrier transport [1–20]. The perovskite and its derivatives have been achieved in various types of solar cell architectures including perovskite-sensitized solar cells, mesoporous (mp)-TiO_2_/perovskite material, and planar p–i–n heterojunction solar cells [21–24]. However, CH_3_NH_3_PbI_3_ perovskite films can be prepared by dual-source thermal evaporation system [25], vapor-assisted solution process [26], and one-step and two-step spin coating procedures for CH_3_NH_3_PbI_3_ formation [27, 28] which has many advantages such as low cost, low temperature, and ease of control.

In this work, we report the solution process fabrication of perovskite solar cells which comprised an architecture CH_3_NH_3_PbI_3_ perovskites formed by a solvent-engineering technology. This study investigated the optical, structural, and surface properties of a perovskite film that is grown on PEDOT:PSS/ITO electrodes by the solvent-engineering technology as functions of thickness in high-performance perovskite solar cells.

## Methods

**Fig. 1:**
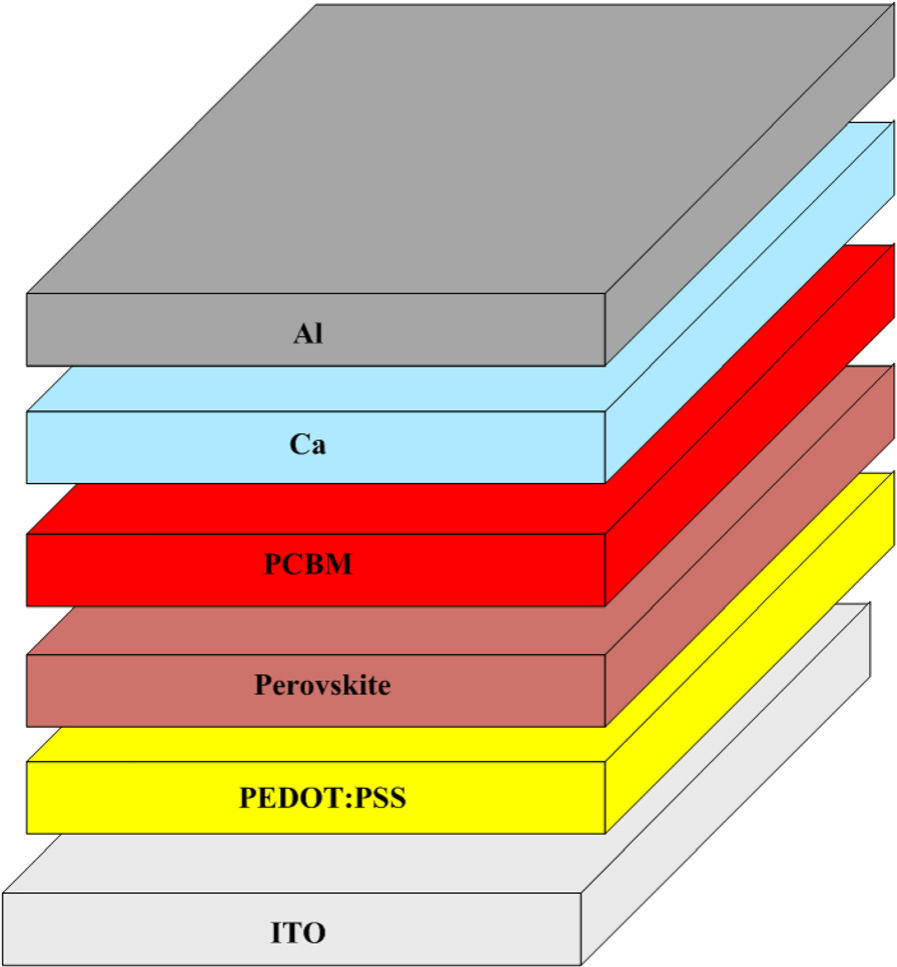
Schematic of the perovskite device configuration consisting of a structure of Al/Ca/perovskite/PEDOT:PSS/ITO substrate

In this study, a PEDOT:PSS (CLEVIOS Al 4083) film was spin-coated on a pre-cleaned ITO substrate at 5000 rpm for 30 s. After spin coating, the film was annealed at 140 °C for 10 min. The perovskite layer was deposited by the solvent-engineering technology of 1.2 M PbI_2_ and 1.2 M methylammonium iodide (MAI) in a cosolvent of dimethyl sulfoxide (DMSO) and γ-butyrolactone (GBL) (vol. ratio = 1:1) in a glove box filled with highly pure nitrogen. The perovskite solutions were then coated onto the PEDOT:PSS/ITO substrate by two consecutive spin coating steps, at 1000 and 5000 rpm for 10 and 20 s, respectively. At 5000 rpm for 20 s, the wet spinning film was quenched by dropping 50 μl of anhydrous toluene. After spin coating, the film was annealed at 100 °C for 10 min. A solution of PCBM was spin-coated on the perovskite layer/PEDOT:PSS/ITO substrate at 3000 rpm for 30 s. Finally, a Ca/Al electrode was completed by thermal deposition with a thickness of 100 nm. Figure 1 schematically depicts the complete structure. The roles of the PCBM film, CH_3_NH_3_PbI_3_ film, and PEDOT:PSS film in the cell structure is electron transport layer, active layer, and hole transport layer, respectively.

A field emission scanning electron microscope (FESEM) (LEO 1530) was used to observe the cross section and surface morphology of the cells. Moreover, the current density–voltage (*J*–*V*) characteristics were measured using a Keithley 2420 programmable source meter under irradiation by a 1000-W xenon lamp. Finally, the irradiation power density on the surface of the sample was calibrated as 1000 W/m^2^.

## Results and Discussion

**Fig. 2:**
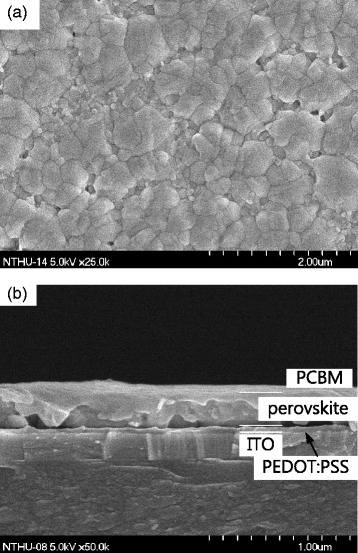
**a** FESEM morphological image of perovskite film. **b** FESEM cross-sectional image showing the device structure

Figure 2a, b presents the cross-sectional and surface FESEM images of the perovskite films on glass substrate. Perovskite prepared by the one-step coating method shows cuboid-like crystals, the average CH_3_NH_3_PbI_3_ crystal size from about 200 nm to about 600 nm, as shown in Fig. 2a. A high-resolution image of the cross section of the obtained perovskite solar cell configuration is shown in Fig. 2b. It clearly indicates the presence of each layer of ITO (200 nm), PEDOT:PSS (~50 nm), perovskite (~200 nm), and PCBM (~80 nm).

**Fig. 3:**
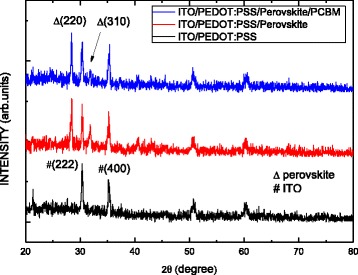
XRD patterns of perovskite and perovskite/PCBM films on ITO/PEDOT:PSS substrates

Figure 3 shows the XRD patterns of CH_3_NH_3_PbI_3_ and PCBM/CH_3_NH_3_PbI_3_ perovskite films deposited on PEDOT:PSS/ITO substrates. The spectra in this study reveal two peaks at the position of 28.39° and 31.86°, which correlate well with (220) and (310) planes of the CH_3_NH_3_PbI_3_ perovskite phase. This result suggests that the solvent in the PCBM film does not destroy the structure of the underlying CH_3_NH_3_PbI_3_ perovskite film during the coating.

**Fig. 4:**
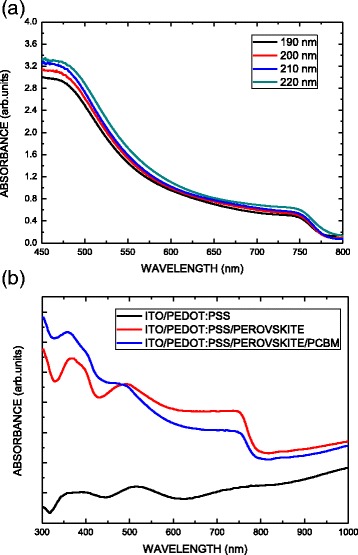
**a** Absorbance spectra of the CH_3_NH_3_PbI_3_ perovskite films with different thicknesses on glass substrates. **b** Absorption spectra of samples with perovskite and PCBM/perovskite films on PEDOT:PSS/ITO substrates

Figure 4 plots the UV-visible absorption measurements. Figure 4a shows the absorbance spectra of the CH_3_NH_3_PbI_3_ perovskite films with different thicknesses on glass substrates. The CH_3_NH_3_PbI_3_ perovskite film with 190-nm thickness is lower than that of the film with 220-nm thickness. In other words, more sunlight can be absorbed to generate excitons in the perovskite film when the thickness increases. Figure 4b shows the absorption spectra of samples with perovskite and PCBM/perovskite films on PEDOT:PSS/ITO substrates. The samples yielded the typical absorption spectrum of CH_3_NH_3_PbI_3_ perovskite between 300 and 760 nm due to the band gap of 1.6 eV [29]. As seen, the absorption of PEDOT:PSS/ITO glass substrate in the figure, in the presence of CH_3_NH_3_PbI_3_, was significantly enhanced throughout the visible region, confirming the possibility of the contribution of CH_3_NH_3_PbI_3_ to the harvesting of light. To compare with the sample of CH_3_NH_3_PbI_3_ on PEDOT:PSS/ITO substrate, for the sample of PCBM/CH_3_NH_3_PbI_3_ on PEDOT:PSS/ITO substrate, the absorption of wavelengths in the range 300–500 nm lightly increases, and the absorption of wavelengths in the range 500–760 nm lightly decreases. That may be attributed to the PCBM absorption [30].

**Fig. 5:**
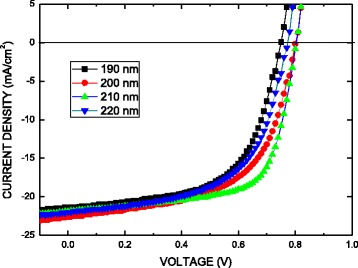
Current–voltage (*J*–*V*) characteristics of perovskite solar cell constructed using the Al/Ca/perovskite/PEDOT:PSS/ITO substrate under a simulated illumination with a light intensity of 100 mW/cm^2^ (AM1.5G)

Figure 5 plots photocurrent *J*–*V* curves of the perovskite solar cell obtained under 100 mW/cm^2^ illumination and the AM1.5G condition. The cell has an active area of 5 × 5 mm^2^ and no antireflective coating. Table 1 lists the main characteristics of those samples. According to Table 1, the series resistance (*R*_s_) of cell increases when the thickness of the CH_3_NH_3_PbI_3_ perovskite film increases, and the thickness of the CH_3_NH_3_PbI_3_ perovskite film can affect the carrier diffusion length in a device that strongly absorbs light in the red spectral region. The perovskite solar cell fabricated on the 210-nm-thick perovskite film showed the highest power conversion efficiency (EFF), *η* = 11.99 % value (*J*_sc_ = 21.9 mA/cm^2^) due to increased photocurrent density. From the *J*–*V* curve and *η* value, we can decide that the optimized passivating thickness of the perovskite film is 210 nm thick. However, further increase in thickness of the perovskite film to 220 nm resulted in decrease of *η* = 9.88 % value (*J*_sc_ = 22 mA/cm^2^). Therefore, a film of optimal thickness would absorb more light and yield a higher current.

**Table 1:** Measurements of all samples in this study

Thickness (nm)	*J*_sc_ (mA/cm^2^)	*V*_oc_ (V)	FF	Efficiency (%)	*R*_s_ (Ω)
190	21.5	0.75	0.595	9.59	3.9
200	22.6	0.81	0.579	10.60	4.4
210	21.9	0.81	0.676	11.99	4.7
220	22.0	0.77	0.583	9.88	4.8

**Fig. 6:**
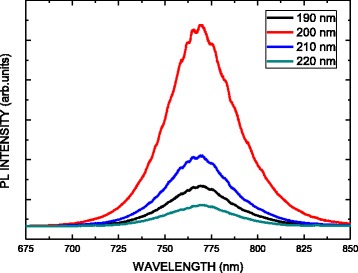
PL spectra of the CH_3_NH_3_PbI_3_ perovskite films with different thicknesses

Figure 6 presents the photoluminescence (PL) spectra of the CH_3_NH_3_PbI_3_ perovskite films with different thicknesses on glass substrates. The dominant peak located at 1.615 eV (768 nm) corresponds to the optical band gap of the CH_3_NH_3_PbI_3_ perovskite films with a direct band gap and can be attributed to the recombination of the near band-to-band (B-B) [29]. When the thickness of the CH_3_NH_3_PbI_3_ perovskite film increases, the PL intensity increases. However, under identical experimental conditions, the PL quantum yield of the 220-nm-thick CH_3_NH_3_PbI_3_ is greatly reduced. Therefore, it was found that a more strikingly quenching effect was in the 220-nm-thick perovskite layer than in the 200-nm-thick perovskite layer.

**Fig. 7:**
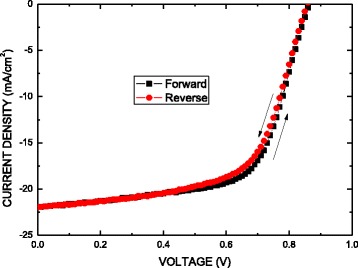
Current–voltage curves with forward and reverse scans for a solar cell showing hysteresis

The hysteresis effect in the perovskite-based solar cells was reported [31–34]. The reasons may include the collection of excess carrier, defects in materials, ion movement caused by polarization, or ferroelectric effects. Figure 7 shows the current–voltage curves with forward and reverse scans for a solar cell showing hysteresis. The scan parameters are scan speed (0.2 V/s) and delay time (40 ms). As shown in Fig. 7, a hysteresis was observed in the *J*–*V* curves of the present cell, 12.04 and 11.52 % for the forward and reverse bias scans. Only a 0.52 % drop in efficiency was observed as compared to that in the forward bias scan. The average values from the *J*–*V* curves in reverse and forward scans exhibited a *J*_sc_ of 21.925 mA/cm^2^, *V*_oc_ of 0.86 V, and FF of 62.5 %, corresponding to a PCE of 11.78 % under standard AM1.5G conditions.

**Fig. 8:**
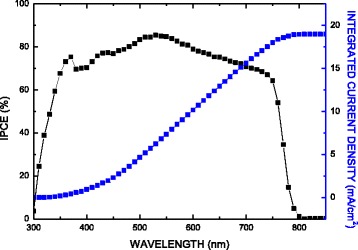
IPCE spectrum of the Al/Ca/perovskite/PEDOT:PSS/ITO substrate (*red squares*). The integrated product of the IPCE spectrum with the AM1.5G photon flux is also shown (*blue squares*)

Figure 8 displays incident photon-to-electron conversion efficiency (IPCE) spectrum of the Al/Ca/perovskite/PEDOT:PSS/ITO substrate (red squares). The integrated product of the IPCE spectrum with the AM1.5G photon flux is also shown (blue squares). The IPCE spectrum shows the expected behavior for a high-performance device based on CH_3_NH_3_PbI_3_. The onset of photocurrent at 800 nm is consistent with the reported band gap of CH_3_NH_3_PbI_3_ [29]. The best device also showed a very broad IPCE plateau of over 80 % between 480 and 600 nm, as shown in Fig. 8. Integrating the product of the AM1.5G photon flux with the IPCE spectrum yields a predicted *J*_sc_ of around 19 mA/cm^2^, which is in agreement with the measured value of around 22 mA/cm^2^.

## Conclusions

High-efficiency and low-cost perovskite/PCBM organic solar cells with various thicknesses of CH_3_NH_3_PbI_3_ perovskite were fabricated. The PCBM film as the electron transport layer in the cell structure can improve the optical absorption in the wavelength range of 300–500 nm, and the absorption in the wavelength range of 500–760 nm is lightly dropped according to the comparison between the samples of PCBM/CH_3_NH_3_PbI_3_ on substrate and CH_3_NH_3_PbI_3_ on substrate. The short-circuit current density and the power conversion efficiency were 21.9 mA/cm^2^ and 11.99 %, respectively, for the optimal measured parameters of the sample with 210-nm-thick CH_3_NH_3_PbI_3_ perovskite.
